# Effect of Surface Finish on Electromigration Reliability of Line-Type Sn-3.0Ag-0.5Cu Solder Joints

**DOI:** 10.3390/ma19173655

**Published:** 2026-08-28

**Authors:** Shuai Meng, Mingliang Huang

**Affiliations:** Electronic Packaging Materials Laboratory, School of Materials Science and Engineering, Dalian University of Technology, Dalian 116024, China

**Keywords:** electromigration, surface finish, line-type solder joint, failure mechanism

## Abstract

The electromigration (EM) reliability of line-type Sn-3.0Ag-0.5Cu (SAC305) solder joints with surface finishes of organic solderability preservatives (OSP), electroless nickel/electroless palladium/immersion Gold (ENEPIG), and Electroplated Ni was systematically investigated under a current density of 1.0 × 10^4^ A/cm^2^ at 150 °C. In the as-soldered state, scallop-shaped Cu_6_Sn_5_ grains formed at both interfaces of the OSP joints. In contrast, driven by the Cu-Ni interaction, (Cu,Ni)_6_Sn_5_ grains formed at both interfaces in the ENEPIG and Electroplated Ni joints, with their morphology varying with Ni content and exhibiting polyhedral, prismatic, or scallop-like shapes. When the surface finishes served as the cathode, the failure rate of the OSP joints (100%) was 1.2 times that of the ENEPIG joints (83%) and 2.0 times that of the Electroplated Ni joints (50%). Extensive dissolution of the Cu substrate caused the rapid failure of the OSP joints, whereas both Ni-P and Ni layers effectively blocked the diffusion of Cu atoms. In the ENEPIG joints, the consumption of Ni atoms transformed the amorphous Ni-P layer into a porous Ni_3_P layer, leading to joint failure. Electroplated Ni possesses a more stable crystalline structure and stronger atomic bonding, so more energy is required to release Ni atoms, resulting in a slower dissolution rate. Compared with the OSP joints, the EM lifetimes of the ENEPIG joints and Electroplated Ni joints were improved by 30% and 53%, respectively.

## 1. Introduction

With the continuous increase in the number of input/outputs (I/Os) on chips, solder joints are downsizing and the current density they carry keeps rising. As a result, the current density through each solder joint can reach up to 10^6^ A/cm^2^, and electromigration (EM) has become a serious reliability issue in high-density packaging [1,2,3,4].

Undergoing EM, the electron wind drives metal atoms to migrate towards the anode. The cathode under bump metallization (UBM) dissolves, while the intermetallic compound (IMC) at the anode interface grows rapidly; this phenomenon is known as the polarity effect [5,6,7,8,9]. To inhibit the dissolution of the cathode Cu substrate, surface finishes such as organic solderability preservative (OSP), electroless nickel/electroless palladium/immersion gold (ENEPIG) and Electroplated Ni are applied to Cu pads to improve the EM reliability of solder joints.

The OSP surface finish is cost-effective, but it dissolves rapidly and exposes the Cu pad during reflow soldering [10,11]. Compared with OSP, the ENEPIG surface finish, as an excellent diffusion barrier, has been widely used in flip-chip solder joints [12]. In the ENEPIG system, the immersion Au layer provides oxidation resistance and improves wettability between the solder and the pad; the electroless Pd layer enhances wettability and acts as a diffusion barrier, as does the electroless Ni-P layer [13]. Ho et al. [14] investigated the EM behavior of ENEPIG/Sn-0.7Cu/ENEPIG and OSP/Sn-0.7Cu/OSP solder joints and found that ENEPIG suppressed Cu pad consumption, thereby improving the EM reliability of solder joints. Kim et al. [15] studied the effect of ENEPIG thickness on the EM behavior of SAC305 solder joints and found that a thicker Ni-P layer significantly improved the EM reliability. Hideaki et al. [16] reported that the diffusion of Ni atoms from the ENEPIG layer into the solder can lead to rapid failure of the solder joints. Zou et al. [17] compared the EM failure modes of OSP and ENEPIG joints and found that OSP joints exhibited rapid Cu pad consumption at the cathode, whereas ENEPIG joints exhibited void and crack formation at the cathode. Hsiao et al. [18] investigated the EM behavior of OSP and ENEPIG joints and found that the (Cu,Ni)_6_Sn_5_ IMC exhibited stronger resistance to EM than the Cu_6_Sn_5_ IMC. Huang et al. [19,20,21] reported the failure mode of Ni-P UBM, in which voids formed and expanded at the cathode interface, the effective cross-sectional area of the solder joint decreased, and more Joule heat was generated locally, ultimately leading to overheating and melting failure. Lee et al. [22] investigated the EM behavior of electroless Ni used as the cathode and observed its localized dissolution under current stress. Compared with ENEPIG, Electroplated Ni offers higher conductivity, greater thermal stability, simpler fabrication processing, and lower cost; therefore, it is considered a more desirable UBM. However, relatively few studies have examined the effect of Electroplated Ni as a UBM on the EM reliability of solder joints. Moreover, previous studies on the influence of surface finishes on the EM reliability mainly focused on microstructural evolution, typically with limited sample sizes. The EM-induced microstructural degradation of solder joints is macroscopically manifested as an increase in electrical resistance, and combining these two metrics offers deeper insight into failure mechanisms. However, the specific influence of surface finishes on the EM failure behavior and lifetime of Sn-3.0Ag-0.5Cu (SAC305) solder joints has rarely been reported and merits systematic investigation.

In the present work, OSP, ENEPIG, and Electroplated Ni were employed as the surface finishes. By combining microstructure characterization with resistance monitoring, the influence of surface finishes on the EM reliability of line-type SAC305 solder joints was comparatively investigated. The current crowding effect and thermomigration (TM) are expected to be avoided in these line-type joints because of their symmetric structure. The Weibull characteristic lifetime under EM was analyzed, and the failure modes of solder joints with different surface finishes were identified. This study provides theoretical guidance for improving the EM reliability of solder joints.

## 2. Materials and Methods

Line-type Cu (OSP/ENEPIG/Electroplated Ni)/SAC305/Cu solder joints were prepared via immersion soldering. First, a 1 μm thick Ni film was electroplated onto 10 mm × 10 mm × 10 mm Cu blocks to serve as a marker layer. Subsequently, Cu films with thicknesses of 60 and 20 μm were electroplated onto this Ni film. The 60 μm Cu films were then coated with OSP and ENEPIG surface finishes, with thicknesses of 0.2 μm and 5 μm, respectively, while the 20 μm Cu film remained uncoated and was used directly as the Cu substrate. Separately, an Electroplated Ni surface finish was prepared by directly depositing a 20 μm thick Ni layer onto a separate Cu block. These prepared Cu blocks-featuring OSP, ENEPIG, and Electroplated Ni finishes-were each mated with the Cu block carrying the bare 20 μm Cu film for subsequent immersion soldering. Flux was applied to the surface finishes, and the interconnect spacing was controlled using a 200 µm diameter stainless steel spacer. Each pair of Cu blocks was then immersed in molten SAC305 solder at 260 °C for 10 s. The soldered assemblies were cross-sectioned into 600 µm × 600 µm specimens by electric discharge machining, and then ground and polished to final dimensions of 300 µm × 300 µm.

**Figure 1:**
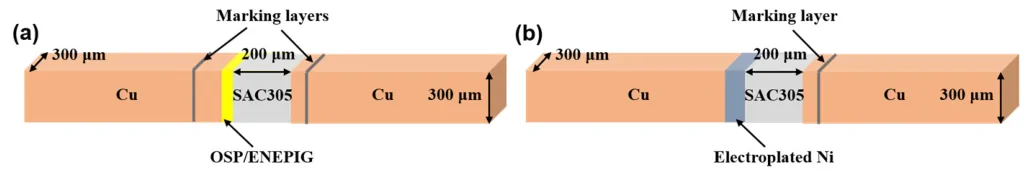
Schematic of line-type solder joints with different surface finishes: (**a**) OSP/ENEPIG and (**b**) Electroplated Ni.

The final cross-sectional area of the line-type solder joints was 300 × 300 μm^2^, and the SAC305 solder thickness between each surface finish (OSP, ENEPIG, and Electroplated Ni) and the Cu substrate was 200 μm, as shown in Figure 1. During EM testing, a constant direct current (DC) was applied to the line-type solder joints using a KEYSIGHT (E36233A, Santa Rosa, CA, USA) power supply. The current density, calculated based on the cross-sectional area of the line-type solder joints, was 1.0 × 10^4^ A/cm^2^. The line-type solder joints were immersed in silicone oil maintained at the target temperature. The actual solder joint temperature was calibrated with a K-type thermocouple and determined to be 150 ± 2 °C. The Kelvin four-point probe method was employed to monitor the electrical resistance of the solder joints in real time during EM, ensuring measurement accuracy. For each surface finish, 24 line-type solder joints were tested: 12 with that finish as the cathode and the other 12 as the anode. All experiments were terminated at 1000 h. The electrical resistance-increase percentage $\alpha \left(t\right)$ over time t is defined as:

(1) $$\alpha (t)=\frac{R(t)-R_{0}}{R_{0}}\times 100\%$$

where R(t) is the time-dependent electrical resistance, and $R_{0}$ is the initial electrical resistance.

After EM testing, the rates of electrical resistance change were statistically analyzed, and the samples were ground and polished for microstructural examination using a scanning electron microscope (SEM, Flex-1000, HITACHI, Tokyo, Japan). Electron probe microanalysis (EPMA, JXA-8530F, JEOL, Tokyo, Japan) was used to identify the phase compositions in the solder joints. The thickness of interfacial IMCs was quantified via Photoshop 2014 image analysis.

## 3. Results and Discussion

**Figure 2:**
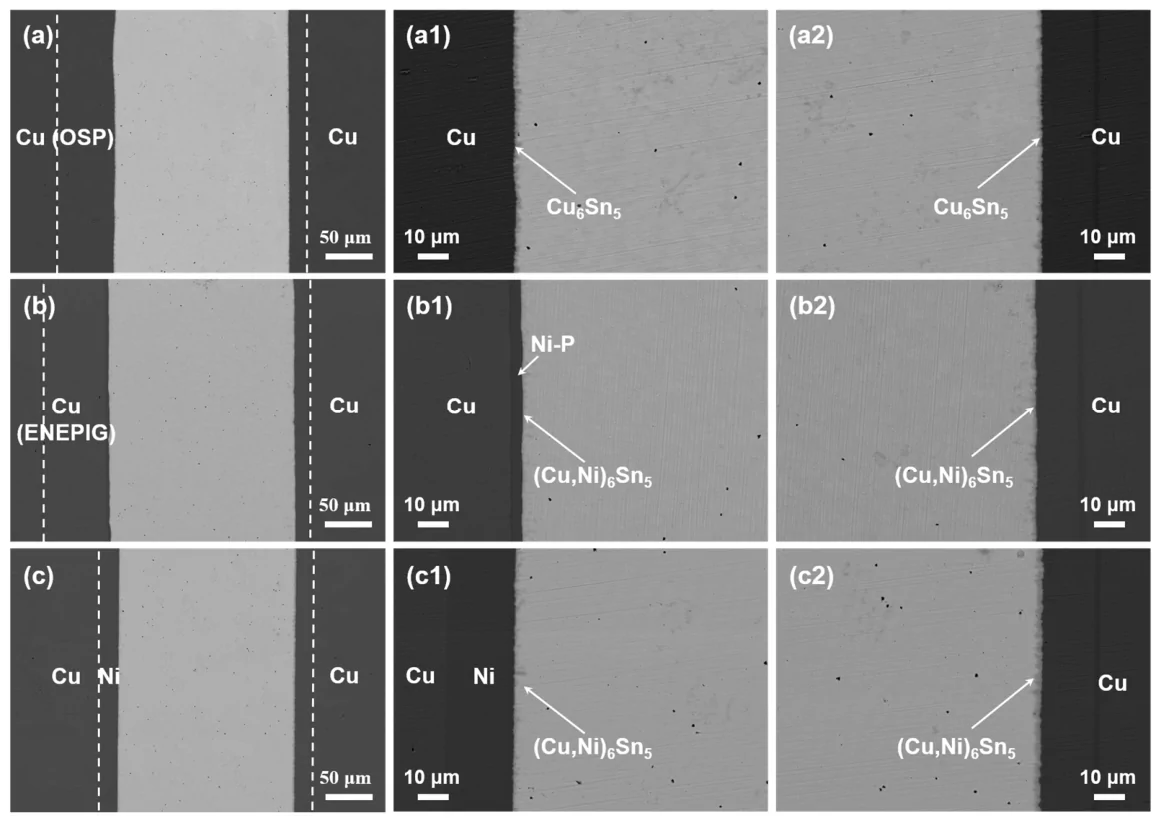
Cross-sectional SEM images of as-soldered line-type solder joints: (**a**–**c**) the entire micrographs, (**a**,**a1**,**a2**) OSP/solder/Cu interconnect, (**b**,**b1**,**b2**) ENEPIG/solder/Cu interconnect and (**c**,**c1**,**c2**) Electroplated Ni/solder/Cu interconnect.

Figure 2 shows the cross-sectional microstructure of the as-soldered line-type solder joints. It was clear that the solder and UBMs were well bonded metallurgically, and no voids were observed in any of the as-soldered specimens. For the OSP surface finish, the thin OSP film completely dissolved into the molten solder during soldering, and Cu_6_Sn_5_ IMCs formed at both Cu/solder interfaces, with thicknesses of 1.05 μm and 1.13 μm, respectively, as shown in Figure 2(a1,a2). For the ENEPIG surface finish, the thin Au and Pd films also completely dissolved into the molten solder during soldering. (Cu,Ni)_6_Sn_5_ IMCs formed at the Ni-P/solder and Cu/solder interfaces, with thicknesses of 0.88 μm and 1.09 μm, respectively, as shown in Figure 2(b1,b2). For the Electroplated Ni surface finish, no significant dissolution was observed, and it remained nearly intact. (Cu,Ni)_6_Sn_5_ IMCs formed at the Ni/solder and Cu/solder interfaces, with thicknesses of 0.79 μm and 1.05 μm, respectively, as shown in Figure 2(c1,c2). Compared with Cu, both Ni-P and Ni significantly inhibited the growth of interfacial IMCs.

**Figure 3:**
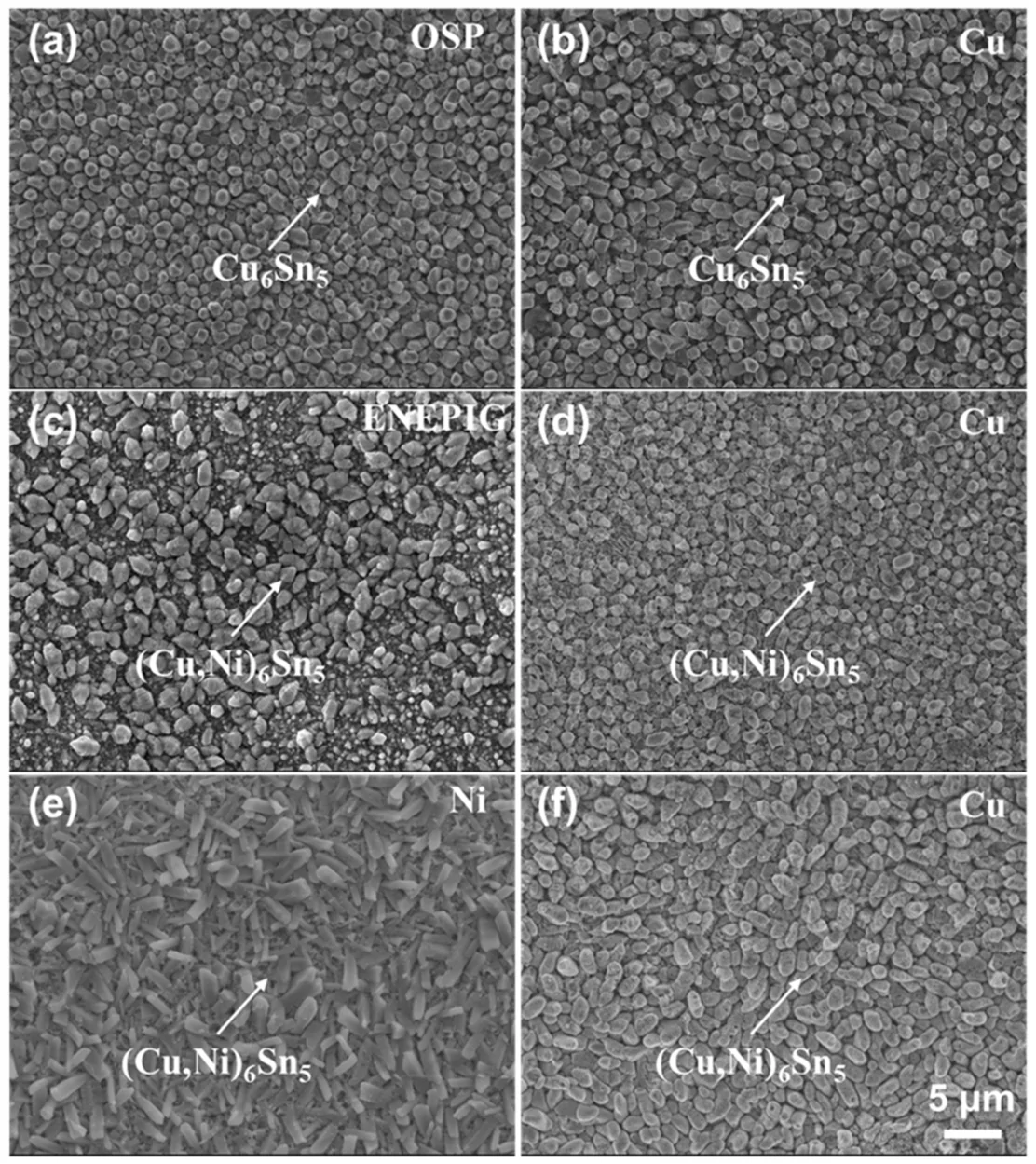
Top-view SEM images of interfacial IMC grains in the as-soldered line-type solder joints: (**a**,**b**) OSP joint, (**c**,**d**) ENEPIG joint and (**e**,**f**) Electroplated Ni joint.

Figure 3 shows the top-view morphologies of the interfacial IMCs. Scallop-shaped Cu_6_Sn_5_ grains formed on both Cu substrates of the OSP joint. Polyhedral-shaped and scallop-shaped (Cu,Ni)_6_Sn_5_ grains formed on the Ni-P and Cu substrates of the ENEPIG joint, respectively, while prismatic-shaped and scallop-shaped (Cu,Ni)_6_Sn_5_ grains formed on the Ni and Cu substrates of the Electroplated Ni joint, respectively. The differences in morphology of (Cu,Ni)_6_Sn_5_ grains may be attributed to the variation in Ni content [23,24], as shown in Table 1.

**Figure 4:**
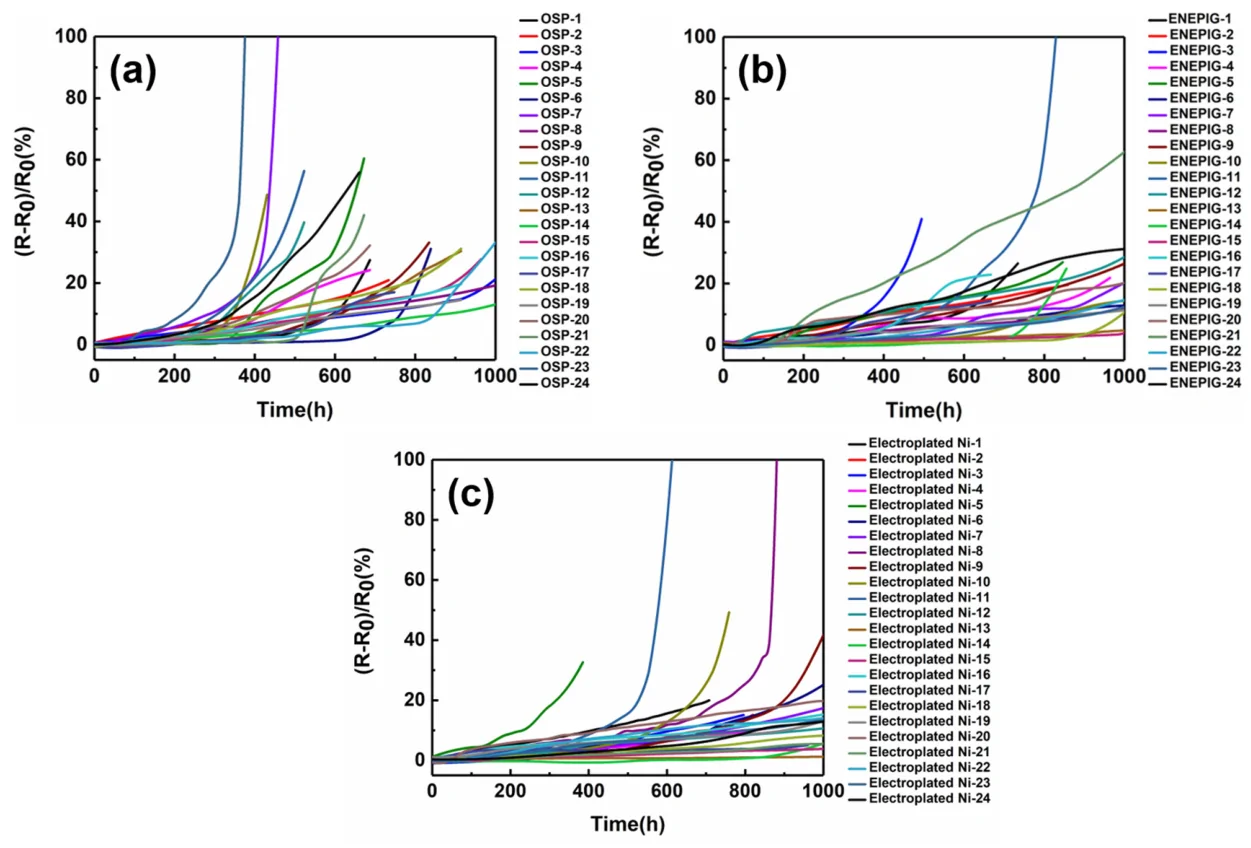
Electrical resistance variations in line-type solder joints as a function of EM time: (**a**) 24 OSP joints, (**b**) 24 ENEPIG joints and (**c**) 24 Electroplated Ni joints.

**Figure 5:**
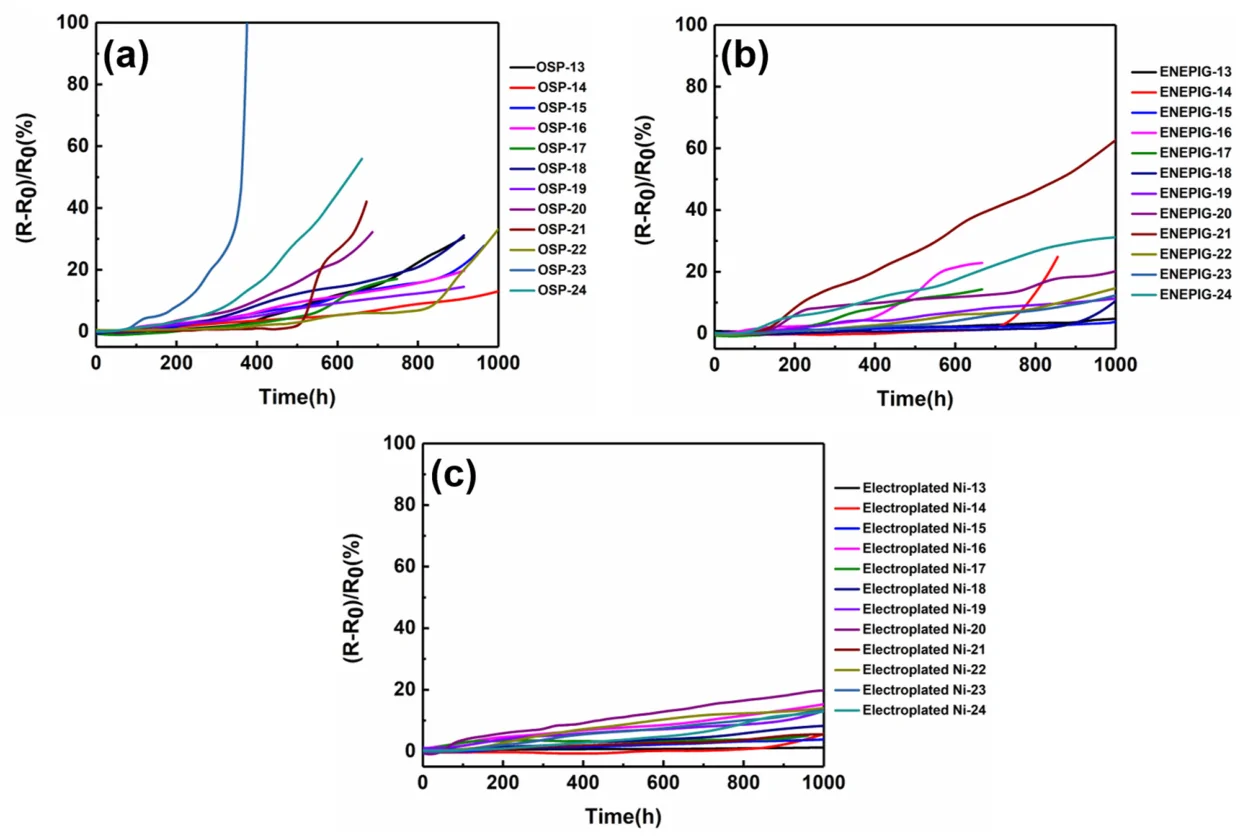
Electrical resistance variations in line-type solder joints as a function of EM time when surface finishes served as the cathode: (**a**) 12 joints with OSP as the cathode, (**b**) 12 joints with ENEPIG as the cathode and (**c**) 12 joints with Electroplated Ni as the cathode.

Figure 4 shows the evolution of electrical resistance of the line-type solder joints as a function of EM time. Overall, the electrical resistance of 24 OSP joints, 24 ENEPIG joints, and 24 Electroplated Ni joints all increased with EM time. A 10% increase in electrical resistance was adopted as the failure criterion. After 1000 h of EM testing, all of 24 OSP joints failed, 22 ENEPIG joints failed and 18 Electroplated Ni joints failed, as shown in Figure 4a, Figure 4b and Figure 4c, respectively. The failure rates of the OSP, ENEPIG and Electroplated Ni joints were 100%, 91.7%, and 75%, respectively. Furthermore, for a more detailed comparison of how the surface finishes affected the EM reliability, the cases in which the surface finishes served as the cathode and as the anode were analyzed separately. When the surface finishes served as the cathode, the OSP joints failed the fastest, with all 12 joints failing. Two of the twelve ENEPIG joints did not fail. For the Electroplated Ni joints, six of the twelve joints exhibited a resistance change below 10% after EM, as shown in Figure 5a–c. The EM failure rate of the OSP joints (100%) was 1.2 times that of the ENEPIG joints (83.3%) and 2.0 times that of the Electroplated Ni joints (50%).

**Figure 6:**
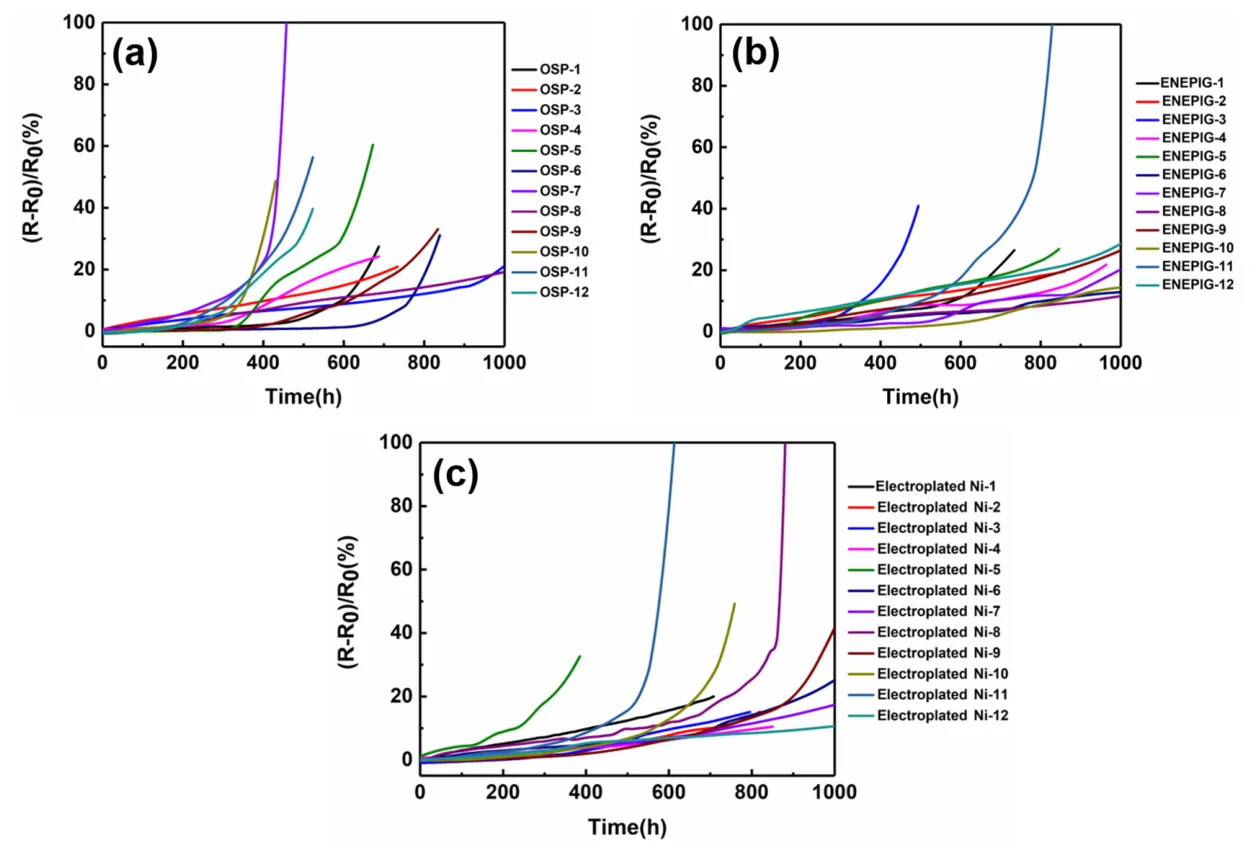
Electrical resistance variations in line-type solder joints as a function of EM time when surface finishes served as the anode: (**a**) 12 joints with OSP as the anode, (**b**) 12 joints with ENEPIG as the anode and (**c**) 12 joints with Electroplated Ni as the anode.

When the surface finishes served as the anode (i.e., the opposite Cu served as the cathode), 12 OSP joints, 12 ENEPIG joints, and 12 electroplated Ni joints all failed. However, compared with OSP joints, the resistance increases in the ENEPIG and Electroplated Ni joints were relatively slow, as shown in Figure 6a–c. As a result, the ENEPIG and Electroplated Ni joints showed better EM reliability than the OSP joints. Notably, when the Electroplated Ni served as the cathode, the joints demonstrated the best resistance to EM.

**Figure 7:**
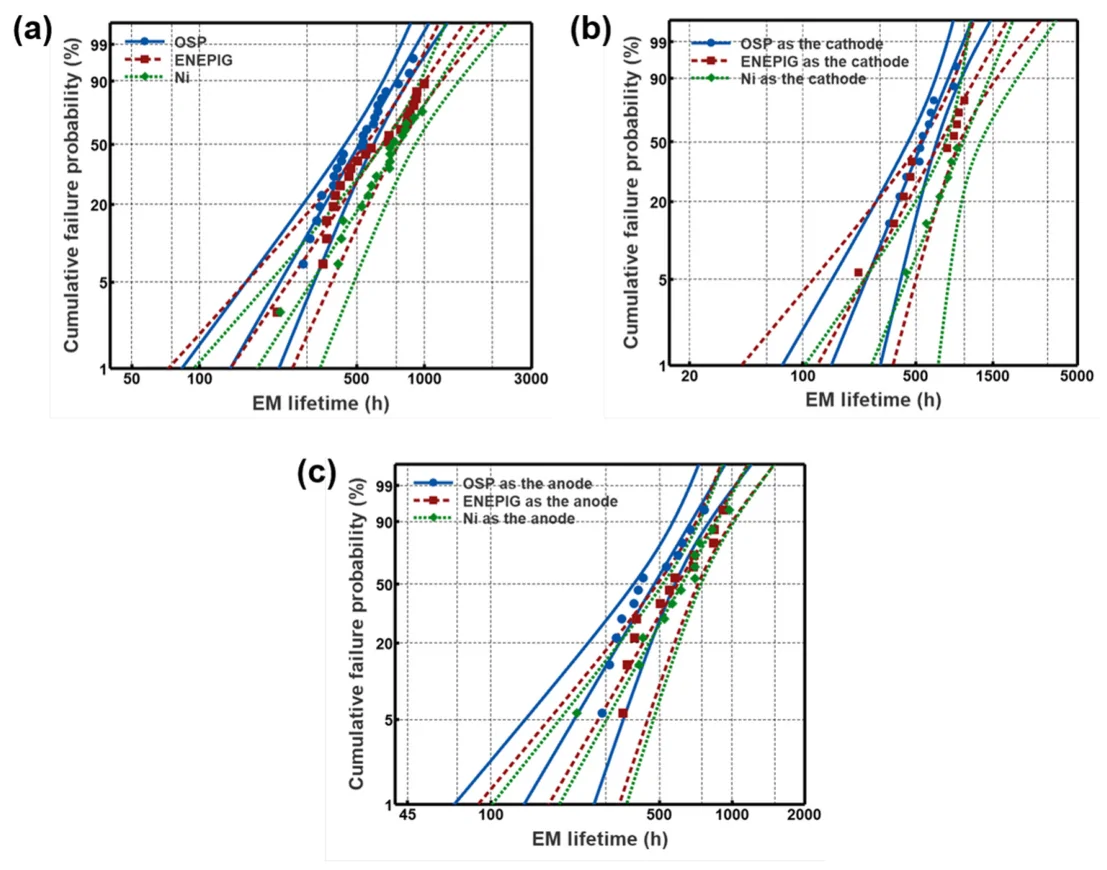
Weibull characteristic EM lifetimes of line-type solder joints: (**a**) 24 joints per surface finish, (**b**) 12 joints per surface finish (surface finishes as the cathode) and (**c**) 12 joints per surface finish (surface finishes as the anode).

Weibull characteristic lifetime analysis was performed on all solder joints, with non-failed ones treated as right-censored (censoring at 1000 h). Figure 7 shows the Weibull plots of cumulative failure probability for line-type solder joints with OSP, ENEPIG and Electroplated Ni surface finishes. The Weibull probability distribution function was expressed as [25]:

(2) $$F(t)=1-\exp[-{\left(\frac{t}{\lambda }\right)}^{k}]$$

where F(t) is the cumulative failure probability, *t* is the time at which the electrical resistance of the solder joints increases by 10%, *k* is the shape parameter, and *λ* is the scale parameter. The *k* and *λ* values reflect the dispersion of the lifetime and the characteristic lifetime of the line-type solder joints, respectively.

Figure 7a shows the Weibull characteristic lifetime analysis results for 24 OSP, 24 ENEPIG, and 24 electroplated Ni joints. The EM Weibull characteristic lifetimes were 572, 741, and 875 h for OSP, ENEPIG and Electroplated Ni solder joints, respectively. The Electroplated Ni joints had the longest characteristic lifetime, followed by the ENEPIG, with the OSP being the shortest. Then, for each surface finish, Weibull characteristic lifetime analysis was performed separately on the 12 solder joints with the surface finish as the cathode and on the 12 solder joints with the surface finish as the anode. When the surface finishes served as the cathode, the Weibull characteristic lifetime of the Electroplated Ni joints (1104 h) was the longest, which was 1.3 times that of the ENEPIG joints (830 h) and 1.8 times that of the OSP joints (615 h), respectively, as shown in Figure 7b. Moreover, six Electroplated Ni joints did not fail, suggesting that their actual lifetime was likely even longer. When the surface finishes served as the anode (i.e., Cu served as the cathode), the EM Weibull characteristic lifetimes were 528, 662, and 686 h for the OSP, ENEPIG and Electroplated Ni joints, respectively, as shown in Figure 7c. The Weibull characteristic lifetime of the Electroplated Ni and ENEPIG joints was comparable and slightly better than that of the OSP joints. The resulting Weibull parameters exhibited good agreement with the corresponding electrical resistance change rate curves. Therefore, the OSP surface finish exhibited the poorest EM resistance, whereas the Electroplated Ni showed the best.

**Figure 8:**
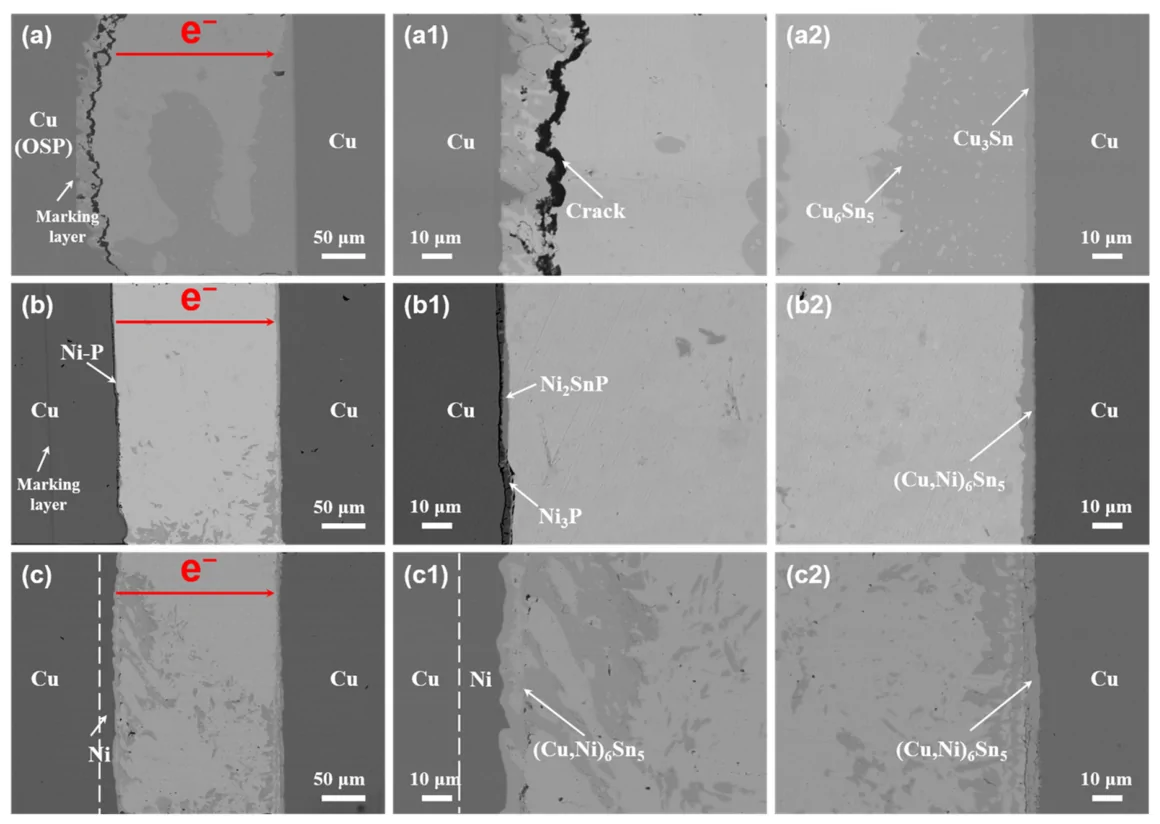
Post-EM cross-sectional SEM images of line-type solder joints with the surface finishes serving as the cathode: (**a**–**c**) the entire micrographs, (**a**,**a1**,**a2**) OSP/solder/Cu interconnect, (**b**,**b1**,**b2**) ENEPIG/solder/Cu interconnect and (**c**,**c1**,**c2**) Electroplated Ni/solder/Cu interconnect.

Figure 8 shows the microstructural evolution of the line-type solder joints that failed the fastest during EM when the surface finishes served as the cathode. An obvious polarity effect was observed in the OSP joint (No. 23), as shown in Figure 8a. Serrated dissolution of the cathode Cu substrate occurred, and massive Cu_6_Sn_5_ IMCs formed at the anode interface and within the solder matrix. The dissolved regions of the Cu substrate were backfilled with solder [26,27,28]. Correspondingly, the consumption of the cathode Cu substrate was about 57.3 μm after about 350 h, and a crack formed at the cathode side, as shown in Figure 8(a1). The fastest dissolution rate of the cathode Cu substrate was 0.16 μm/h. The Cu_6_Sn_5_ and Cu_3_Sn layers at the anode had thicknesses of 44.75 μm and 3.17 μm, respectively, as shown in Figure 8(a2). With the gradual dissolution of the cathode Cu substrate, the crack propagated across the entire cathode interface, causing EM failure of the OSP joint. Figure 8b shows the microstructure of the ENEPIG joint (No. 21) after EM. The cathode Cu substrate beneath the Ni-P layer did not dissolve, and the (Cu,Ni)_6_Sn_5_ IMC at the anode interface became slightly thicker. Further observation revealed that the cathode Ni-P layer (5 μm) had been completely consumed and transformed into a porous Ni_3_P layer after 1000 h. The fastest dissolution rate of the cathode Ni-P layer was 0.005 μm/h. Moreover, a thin Ni_2_SnP layer formed at the interface between the solder and Ni_3_P, as shown in Figure 8(b1). The thickness of the (Cu,Ni)_6_Sn_5_ IMC at the anode interface was about 4.12 μm, as shown in Figure 8(b2). The complete consumption of the Ni-P layer and its transformation into porous Ni_3_P led to EM failure of the ENEPIG joint. Similar to the Cu substrate, the dissolution of the Ni layer led to the EM failure of the Electroplated Ni joint (No. 20), but at a significantly slower rate, as shown in Figure 8c. The consumption of the Ni layer was only 3.13 μm after 1000 h, and the (Cu,Ni)_6_Sn_5_ IMCs at both the cathode and anode interfaces were slightly thicker. The fastest dissolution rate of the cathode Ni layer was 0.003 μm/h. The thicknesses of the (Cu,Ni)_6_Sn_5_ IMCs at the cathode and anode interfaces were 4.53 μm and 4.74 μm, respectively, as shown in Figure 8(c1,c2). A significant amount of precipitated IMC was also observed in the solder matrix. Nevertheless, no cracks or other defects were observed in the solder joint. The dissolution rates were calculated as the cathode UBM consumption of the fastest-failing samples divided by their individual test times (Cu: 0.16 μm/h, Ni-P: 0.005 μm/h, Ni: 0.003 μm/h). Consequently, the Electroplated Ni exhibited superior EM resistance compared to both the OSP and ENEPIG surface finishes.

**Figure 9:**
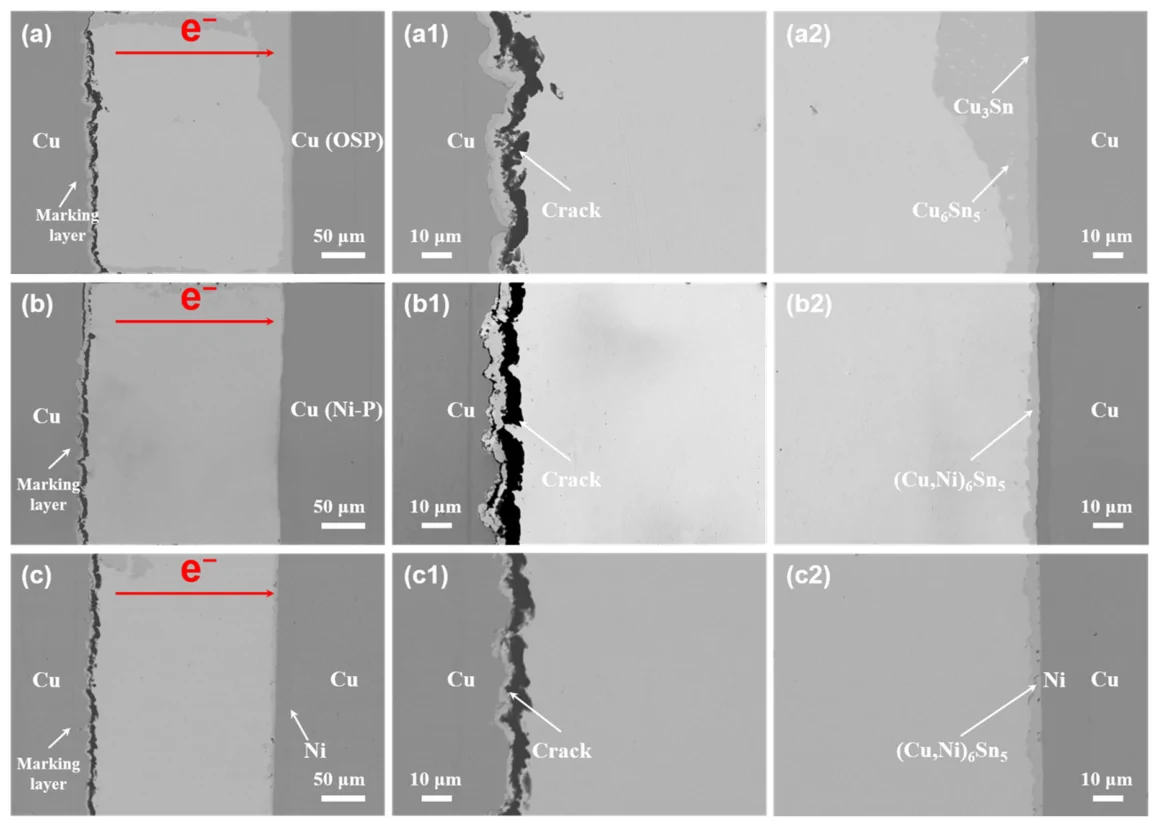
Post-EM cross-sectional SEM images of line-type solder joints with the surface finishes serving as the anode: (**a**–**c**) the entire micrographs, (**a**,**a1**,**a2**) OSP/solder/Cu interconnect, (**b**,**b1**,**b2**) ENEPIG/solder/Cu interconnect and (**c**,**c1**,**c2**) Electroplated Ni/solder/Cu interconnect.

Figure 9 shows the microstructural evolution of the line-type solder joints after EM when the surface finishes served as the anode. A distinct polarity effect was observable in all solder joints. Extensive dissolution occurred on the cathode Cu substrate of all solder joints, leading to the formation of cracks at the cathode interface. These cracks then propagated across the entire interface, ultimately resulting in failure of solder joints, as shown in Figure 9(a1,b1,c1). Meanwhile, the IMCs at the anode interface of the OSP, ENEPIG, and Electroplated Ni joints thickened and transformed into continuous layers, with thicknesses of 19.61, 2.32, and 3.41 μm, respectively, as shown in Figure 9(a2,b2,c2). Compared with OSP joints, the ENEPIG and Electroplated Ni joints exhibited a thinner IMC layer at the anode interface, indicating reduced dissolution of the cathode Cu substrate. This is because (Cu,Ni)_6_Sn_5_ IMC exhibited better EM resistance than Cu_6_Sn_5_ IMC. However, when Cu served as the cathode, the solder joints showed poorer EM resistance than when ENEPIG or Electroplated Ni served as the cathode. The failure mode in all those solder joints was characterized by extensive dissolution of the Cu substrate at the cathode.

In summary, both the ENEPIG and Electroplated Ni surface finishes effectively suppressed the dissolution of the cathode Cu substrate, thereby improving the EM reliability of solder joints. Between the two, Electroplated Ni exhibited better EM resistance.

**Figure 10:**
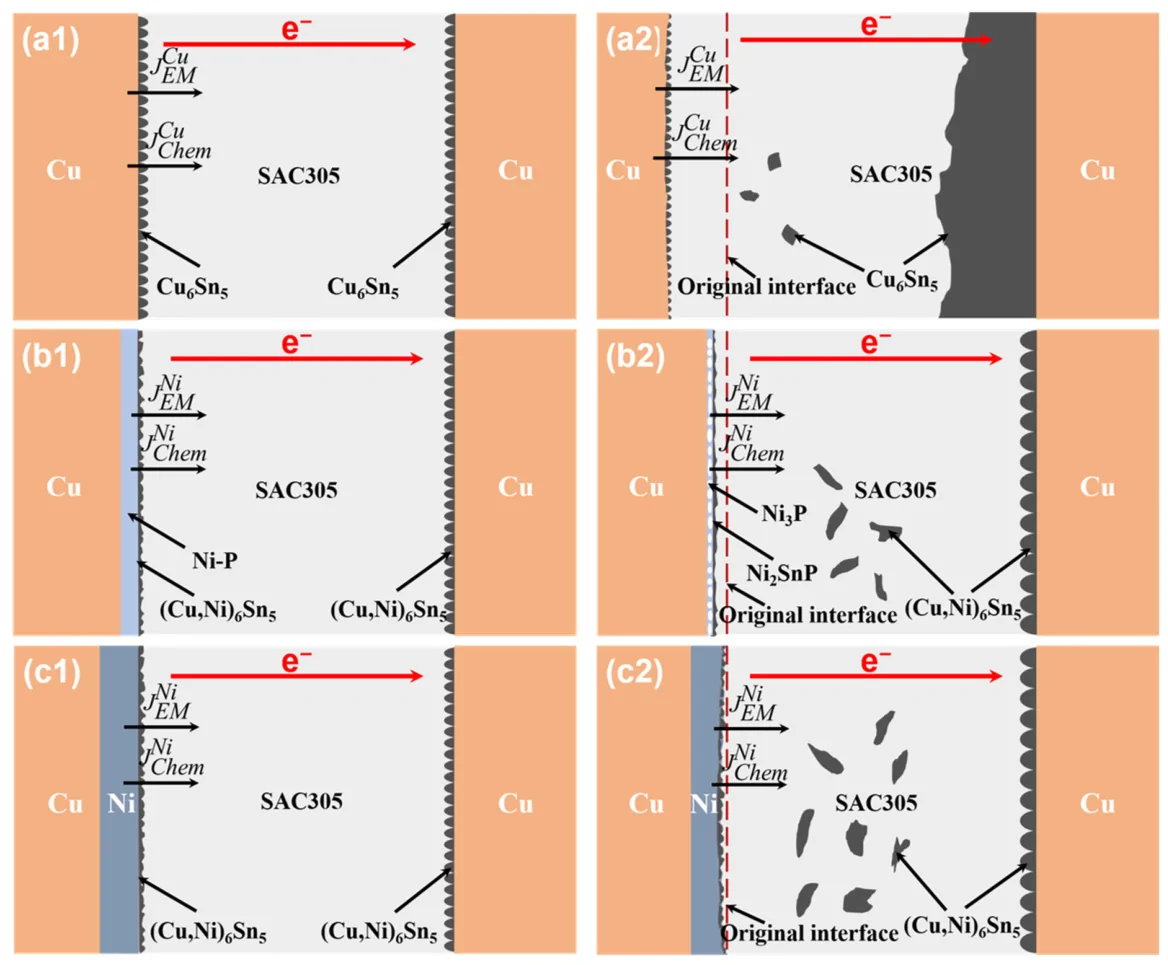
Schematics of failure mechanisms of line-type solder joints with the surface finishes serving as the cathode: (**a1**,**a2**) OSP joint, (**b1**,**b2**) ENEPIG joint, (**c1**,**c2**) Electroplated Ni joint, (**a1,b1,c1**) before EM and (**a2,b2,c2**) after EM.

Figure 10 shows the schematic of the failure mechanisms of the line-type solder joints with the surface finishes serving as the cathode. $J_{\mathrm{chem}}$ is the diffusion term due to the chemical potential gradient, and $J_{\mathrm{em}}$ is the diffusion term due to electron wind force. Under the chemical potential gradient, the atomic diffusion flux can be expressed as follows:

(3) $$J_{\mathrm{chem}}=D\frac{\partial C}{\partial X}$$

Under the electron wind force, the atomic diffusion flux can be expressed as follows:

(4) $$J_{\mathrm{em}}=C\frac{D}{kT}Z^{*}e\rho j$$

where *C* is the concentration, *D* is the diffusivity, k is Boltzmann’s constant, *T* is the temperature, Z* is the effective charge number, e is the electron charge, ρ is the resistance, and *j* is the current density.

The electrons flowed from the OSP (Cu) to the opposite Cu; that is, the Cu atoms of the cathode Cu substrate experienced downwind diffusion. The flux of Cu atoms induced by the chemical potential gradient was in the same direction as that induced by the electron wind force, as shown in Figure 10(a1). The total flux of Cu atoms at the cathode Cu/solder interface in the direction of electron flow was as follows:

(5) $$J_{\mathrm{Cu}}^{\mathrm{total}}=J_{\mathrm{chem}}^{\mathrm{Cu}}+J_{\mathrm{em}}^{\mathrm{Cu}}$$

As a result, the diffusion of Cu atoms from the cathode Cu substrate into the solder was accelerated during EM. As the EM time increased, the cathode Cu substrate underwent excessive dissolution, leading to crack formation at the cathode interface, while the Cu atoms that diffused to the anode precipitated as Cu_6_Sn_5_ IMC at the anode interface and within the solder matrix. The dissolution of the cathode Cu substrate and the crack propagation eventually led to the EM failure of the OSP joint, as shown in Figure 10(a2).

Similarly, when ENEPIG served as the cathode, the Ni atoms in the Ni-P layer were driven away from the cathode not only by the chemical potential gradient but also by the electron wind force, as shown in Figure 10(b1). The total flux of Ni atoms at the Ni-P/solder interface in the direction of electron flow was expressed as:

(6) $$J_{\mathrm{Ni}}^{\mathrm{total}}=J_{\mathrm{chem}}^{\mathrm{Ni}}+J_{\mathrm{em}}^{\mathrm{Ni}}$$

As the Ni atoms diffused out of the Ni-P layer, the P atoms remained and thus accumulated in the original Ni-P layer, forming a Ni_3_P layer. The interfacial reaction is expressed as:

Ni_85_P_15_ → 15Ni_3_P + 40Ni (7)

Moreover, when the Sn atoms reached the Ni_3_P/solder interface, Sn reacted with the Ni_3_P layer to form a Ni_2_SnP layer between the Ni_3_P and solder. The interfacial reaction between Ni_3_P and Sn is expressed as follows:

Ni_3_P + Sn → Ni_2_SnP + 40Ni (8)

As the EM time increased, the Ni-P layer was completely consumed and transformed into a porous Ni_3_P structure, which led to the EM failure of the ENEPIG joint, as shown in Figure 10(b2).

For the Electroplated Ni joint, the total flux of Ni atoms at the Electroplated Ni/solder interface was similar to that at the Ni-P/solder interface, as shown in Equation (6). Under EM, the cathode Ni layer also dissolved, but the degree of dissolution was significantly reduced, as shown in Figure 10(c1,c2).

**Figure 11:**
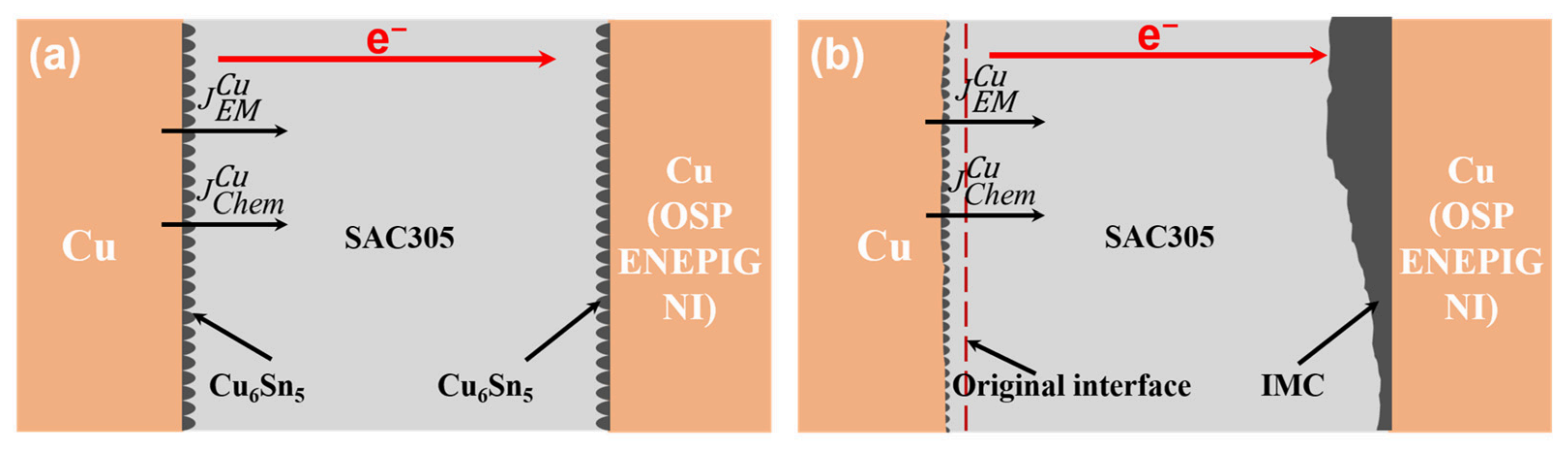
Schematics of the failure mechanism of line-type solder joints with the surface finishes serving as the anode: (**a**) before EM and (**b**) after EM.

Figure 11 shows the schematic of the failure mechanisms of the line-type solder joints with the surface finishes serving as the anode. The total flux of Cu atoms at the Cu/solder interface of OSP, ENEPIG and Electroplated Ni joints was similar to that at the OSP (Cu)/solder interface, as shown in Equation (5). Under the electron wind, a large number of Cu atoms migrated directionally from the cathode Cu substrate to the anode. Without the protection of ENEPIG or Electroplated Ni, the Cu substrate at the cathode of all solder joints dissolved rapidly, leading to crack formation at the cathode interface and final failure.

ENEPIG and Electroplated Ni could effectively protect the Cu substrate from dissolution, thereby enhancing the EM reliability of the solder joints. Ni-P is an amorphous substance, and this non-equilibrium state is unstable and tends to transform into a crystalline state at high temperature. Under EM, the Ni atoms in Ni-P were released, thereby transforming it into the stable crystalline state Ni_3_P. The Ni_3_P layer has a porous structure but still protects the cathode Cu substrate from dissolution. Although the initial thicknesses of both the Electroplated Ni and Ni-P layers may influence the EM lifetime of joints, after 1000 h of testing, the Ni layer exhibited only 3.13 μm of consumption, whereas the Ni-P layer showed 5 μm, indicating that the Electroplated Ni has superior EM resistance compared to the Ni-P layer. Although Electroplated Ni also experienced dissolution, its dissolution rate was significantly slower than that of Cu. This is attributed to the fact that Ni has a significantly lower solubility in Sn than Cu at 150 °C. Under current stressing, Cu atoms enter the solder and migrate towards the anode, thereby sustaining the continuous dissolution of the cathodic Cu UBM. In contrast, once Ni atoms enter the solder, the solder rapidly becomes saturated, causing the flux of Ni atoms to decrease sharply and thereby suppressing the dissolution of the Ni UBM. Moreover, it is well known that the higher the bonding strength between metal atoms, the greater the energy required to break the metallic bond, and the more difficult it is for the metal atoms to escape. The elastic moduli of Ni and Cu are 207 GPa and 119 GPa, respectively. From the perspective of microstructure, the elastic modulus is closely related to the bonding strength of atomic pairs [29]. The melting points of Ni and Cu are 1453 °C and 1083.4 °C, respectively. Typically, the higher the melting point, the stronger the metallic bonds. Therefore, more energy is required to release Ni atoms from the Electroplated Ni layer. Furthermore, (Cu,Ni)_6_Sn_5_ IMC exhibits higher thermal stability owing to Ni doping, rendering it less prone to dissolution during EM [30,31]. (Cu,Ni)_6_Sn_5_ IMCs formed at both interfaces of the ENEPIG and Electroplated Ni joints, whereas Cu_6_Sn_5_ IMCs formed at both interfaces of the OSP joints. Therefore, the ENEPIG and Electroplated Ni joints exhibited better EM resistance, with Electroplated Ni providing the most significant improvement in EM reliability.

## 4. Conclusions

1. In the as-soldered state, scallop-shaped Cu_6_Sn_5_ grains formed at both Cu/solder interfaces of the OSP joints; in the ENEPIG joints, polyhedral-shaped (Cu,Ni)_6_Sn_5_ grains formed at the Ni-P/solder interface while scallop-shaped (Cu,Ni)_6_Sn_5_ grains formed at the Cu/solder interface; in the Electroplated Ni joints, prismatic-shaped (Cu,Ni)_6_Sn_5_ grains formed at the Ni/solder interface and scallop-shaped (Cu,Ni)_6_Sn_5_ grains formed at the Cu/solder interface.
2. When the surface finishes served as the cathode, the failure mode was that the dissolution and depletion of the cathodes. The fastest dissolution rate of the cathode Cu substrate in the OSP joints was 0.16 μm/h, whereas that of the cathode Ni-P layer in the ENEPIG joints was 0.005 μm/h, and that of the cathode Ni layer in the Electroplated Ni joints was only 0.003 μm/h; consequently, the failure rate of the OSP joints (100%) was 1.2 times that of the ENEPIG joints (83%) and 2.0 times that of the Electroplated Ni joints (50%).
3. Compared with Ni-P and Cu, Electroplated Ni possesses a more stable crystalline structure and stronger atomic bonding. Consequently, the higher energy barrier for releasing Ni atoms from the Electroplated Ni layer substantially enhances the EM reliability of solder joints.
4. When the surface finishes served as the anode, the failure mode was that the cathode Cu substrate underwent extensive dissolution in all solder joints, leading to crack formation at the cathode interface and, ultimately, EM failure.
5. Owing to the Cu-Ni interaction, the (Cu,Ni)_6_Sn_5_ IMCs formed in the ENEPIG and Electroplated Ni joints exhibited higher thermal stability than the Cu_6_Sn_5_ IMCs in the OSP joints, making them more resistant to decomposition under EM. Compared with the OSP joints, the EM lifetimes of the ENEPIG joints and Electroplated Ni joints were improved by 30% and 53%, respectively.

## Figures and Tables

**Table 1:** Compositions of interfacial IMCs in line-type solder joints with different surface finishes.

	OSP Joint	ENEPIG Joint	Electroplated Ni Joint
(OSP/ENEPIG/Ni)/solder	Cu_6_Sn_5_	(Cu_0.61_,Ni_0.39_)_6_Sn_5_	(Cu_0.69_,Ni_0.31_)_6_Sn_5_
Cu/solder	Cu_6_Sn_5_	(Cu_0.96_,Ni_0.04_)_6_Sn_5_	(Cu_0.99_,Ni_0.01_)_6_Sn_5_

## Data Availability

The original contributions presented in this study are included in the article. Further inquiries can be directed to the corresponding author.
